# Incidental placental choriocarcinoma in a term pregnancy: a case report

**DOI:** 10.1186/1752-1947-2-330

**Published:** 2008-10-16

**Authors:** Christopher Chung, Ming-Shian Kao, Deborah Gersell

**Affiliations:** 1 Department of Obstetrics and Gynecology, St. Louis University, 6420 Clayton Road, Suite 290, St. Louis, Missouri 63117, USA; 2 Division of Gynecologic Oncology, Department of Obstetrics and Gynecology, St. Louis University, 6420 Clayton Road, Suite 290, St. Louis, Missouri 63117, USA; 3 Department of Pathology, St. John's Mercy Medical Center, 615 South New Ballas Road, St. Louis, Missouri 63141, USA

## Abstract

**Introduction:**

Gestational choriocarcinoma occurs in 1 in 40,000 pregnancies. Of all forms of gestational choriocarcinoma, placental choriocarcinoma is the most rare. Maternal choriocarcinoma is usually diagnosed in symptomatic patients with metastases. The incidental finding of a choriocarcinoma confined to the placenta with no evidence of dissemination to the mother, or infant is the least common scenario.

**Case presentation:**

The patient is an 18 year-old Gravida 1 Para 1 African American female who delivered a viable 3641 g female infant at 39 weeks gestation. Her pregnancy course was complicated by gestational hypertension during the third trimester. Her placenta revealed intraplacental choriocarcinoma. She was then followed closely by the Gynecologic Oncology service with a weekly serum beta human chorionic gonadotropin value. Beta human chorionic gonadotropin values dropped from 3070 mIU/ml to less than 2 mIU/ml two months post partum. No chemotherapy was initiated. Metastasis was ruled out by chest x-ray and whole body computed tomography scan. To date, both mother and baby are well.

**Conclusion:**

Due to the potential fatal outcome of placental choriocarcinoma, careful evaluation of both mother and infant after the diagnosis is made is important. The incidence of placental choriocarcinoma may actually be higher than expected since it is not routine practice to send placentas for pathological evaluation after a normal spontaneous delivery. The obstetrician, pathologist, and pediatrician should have an increased awareness of placental choriocarcinoma and its manifestations.

## Introduction

Gestational choriocarcinoma occurs in 1 in 40,000 pregnancies. It is a highly aggressive malignant tumor of the trophoblasts found in association with any form of gestation. Of all forms of gestational choriocarcinoma, placental choriocarcinoma is the most rare and is usually diagnosed in symptomatic patients with metastases. Metastases to the lung and brain usually occur in the mother, but metastatic choriocarcinoma in the fetus or neonates does occur. The incidental finding of a choriocarcinoma confined to the placenta with no evidence of dissemination to mother or infant is the least common scenario.

## Case presentation

**Figure 1:**
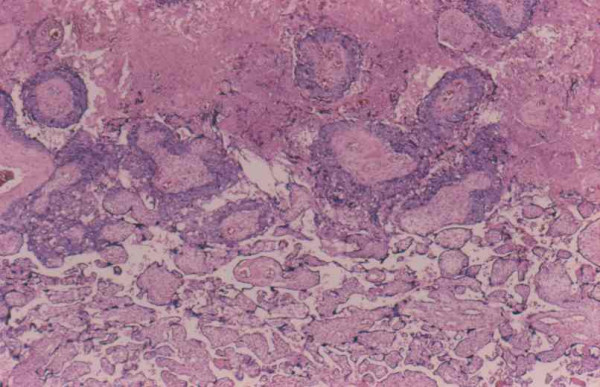
Interface between choriocarcinoma with central necrosis (top) and normal placenta (hematoxilin and eosin staining).

**Figure 2:**
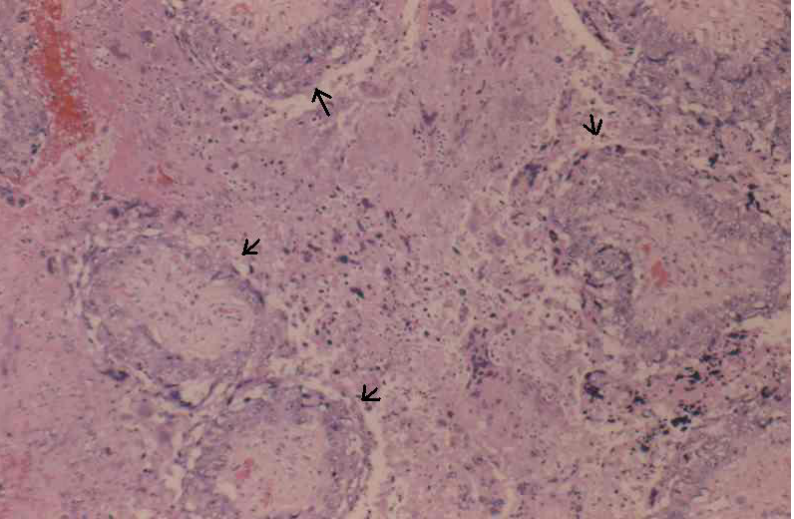
Villi surrounded by collars of neoplastic trophoblast (arrows).

The patient is an 18 year-old African American Gravida 1 Para 0 at her 39^th ^weeks of gestation who presented to a hospital in the St. Louis area in early labor. After rupture of membranes, light meconium was noted. The patient subsequently delivered a viable female infant vaginally, weighing 3641 g, with Apgar scores of 9 and 9 at 1 and 5 minutes. Her pregnancy course was complicated by gestational hypertension during the third trimester. She was treated with magnesium sulfate during labor for possible pre-eclampsia due to elevated blood pressure intrapartum. Her blood pressure was normal after delivery, and her post partum course was unremarkable. The placenta appeared to be grossly normal with a 3 vessel cord at the time of delivery. Because of the patient's history of gestational hypertension, the placenta was sent to pathology and placental choriocarcinoma was diagnosed (Figures 1 &2). She was then referred to and followed closely by the Gynecologic Oncology service. Her serum beta human chorionic gonadotropin (hCG) dropped from 3070 mIU/ml to less than 2 mIU/ml six weeks post partum. No chemotherapy was initiated. She had also undergone multiple studies such as chest x-ray and computed tomography (CT) scan which all ruled out metastasis during the one year post partum period. The infant was followed by the pediatric service. To date, both mother and baby have been disease free for eight years.

## Pathologic findings

The placenta measured 15 × 16 × up to 4 cm with a trimmed weight of 530 grams and was notable only for a 3 cm cyst-like area on cut section. Microscopic examination showed a choriocarcinoma with a biphasic proliferation of atypical and mitotically active cytotrophoblast and syncytiotrophoblast notable for extensive involvement of villi, many completely encompassed by collars of neoplastic trophoblast. Some villi were partially involved with a transition from normal to neoplastic trophoblast. There was no involvement of villous stromal vessels. The tumor showed extensive central necrosis in which the ghost-like outlines of necrotic villi could be discerned. The remainder of the placenta was mature, showing only focal villous edema.

## Discussion

We performed a medline search (1966 to 2008) using the keywords *placenta, choriocarcinoma*, and *trophoblastic disease*. We found 21 articles with 32 cases of placental choriocarcinoma in the English literature. Fourteen cases of choriocarcinoma were confined to the placenta with no evidence of dissemination to mother or infant; five of these 14 cases were incidentally identified in patients with normal pregnancies. To our knowledge, our case is the sixth of this type.

Choriocarcinoma in the placenta differs somewhat from those occurring elsewhere. The majority of placental choriocarcinomas appeared as poorly defined lesions macroscopically, and they are often interpreted as placental infarcts or intervillus thrombi. It is often only after metastatic symptoms occur in mother or infant that the placenta is resubmitted for further evaluation, and small lesions are identified microscopically. When recognized grossly, most placental choriocarcinomas have been described as yellow-white granular lesions thought to be infarcts. Microscopically, placental choriocarcinomas usually revealed areas in which the trophoblast is distributed as a mantle around villi with partial involvement and transition from normal trophoblast. This surface villous growth is often at the periphery of a central zone composed of necrotic, confluent trophoblast and villi explaining its gross resemblance to an infarct. Invasion of villous stroma has been described in rare cases.

Obstetrically, the majority of the reported cases had a normal prenatal course. Placental choriocarcinoma was often diagnosed after the mother developed symptoms of metastasis either antepartum or postpartum. The symptoms included vaginal bleeding, chest pain, and neurological signs such as seizures and stroke. In terms of maternal outcome, mothers who developed metastasis were often treated with chemotherapy which consisted of etoposide, methotrexate, actinomycin-D, cytoxan or vincristine. The response rate after chemotherapy is high; the majority of mothers were disease free after 1 year. Four mothers died of disease, but these 4 cases all occurred in the early 1980s [1,2]. Kodama et al reported a case where the mother underwent partial lobectomy after pulmonary lesions were identified in 1994 [3]. Four cases of hysterectomy were reported by Brewer et al in 1981, Ollendorf in 1990, and Flam in 1996 [1,4,5]. In mothers with no evidence of metastasis, only three of the 14 cases were treated with chemotherapy; the others were followed with serial serum hCG measurement until it dropped to zero during the post partum course. Multiple imaging modalities such as X-ray, CT scan and magnetic resonance imaging were often used to rule out metastasis. Our patient continued to have annual follow-up with the Gynecologic Oncology service after multiple studies to rule out metastasis in the first year post partum.

In terms of fetal outcome, ten cases resulted in either intrauterine demise or neonatal death. In 2006, Liu and Guo reported the only case in which an infant was successfully treated with chemotherapy after metastasis to jejunum and lungs was identified [6]. Most of the infants born showed no evidence of disease after 1 year.

Due to the potential fatal outcome of placental choriocarcinoma, the diagnosis should require careful evaluation of both mother and infant. Other clinical manifestations such as hyperthyroidism which is sometimes associated with high serum HCG should also be ruled out. Hyperemesis gravidarum is a common symptom in patients with hyperthyroidism. Serial serum beta hCGs and imaging studies should be employed to rule out metastasis. Maternal metastasis is very responsive to chemotherapy. Fetal metastasis is rare and usually fatal. In cases where placental choriocarcinoma is confined to the placenta without evidence of metastasis, only three of the 14 cases were treated with methotrexate chemotherapy, and the others were followed by serial beta hCG and imaging studies, all with excellent outcome. We believe in these cases, no chemotherapy is needed, providing serial beta hCG and imaging studies during postpartum follow up remain normal.

## Conclusion

It is not a routine practice to send a placenta for pathological evaluation following a normal spontaneous delivery. However, a number of cases, like ours, reported incidental findings of placental choriocarcinoma in asymptomatic mothers and infants with no evidence of metastases. In the majority of these cases, the placenta was sent to pathology due to other pregnancy complications such as intrauterine growth restriction, pre-eclampsia, maternal fetal hemorrhage, and in our case, gestational hypertension. It is our belief that the incidence of placental choriocarcinoma may actually be higher than reported. It is also well established that early detection and treatment of gestational trophoblastic disease improves treatment outcome. The obstetrician, pathologist and pediatrician should have an increased awareness of placental choriocarcinoma and its manifestations. Clinical suspicion and any gross placental anomaly should mandate a thorough pathological examination of the placenta.

## Abbreviations

hCG: human chorionic gonadotropin; CT: computed tomography.

## Competing interests

The authors declare that they have no competing interests.

## Authors' contributions

CC gathered the data, searched the literature and drafted the manuscript. DG examined the placenta, searched the literature and drafted the manuscript. MSK treated the patient, searched the literature and helped to draft the manuscript. All authors read and approved the final manuscript.

## Consent

Written informed consent was obtained from the patient for publication of this case report and accompanying images. A copy of the written consent is available for review by the Editor-in-Chief of this journal.
